# A Biodegradable Polyethylenimine-Based Vector Modified by Trifunctional Peptide R18 for Enhancing Gene Transfection Efficiency In Vivo

**DOI:** 10.1371/journal.pone.0166673

**Published:** 2016-12-09

**Authors:** Jing Hu, Manman Zhu, Kehai Liu, Hua Fan, Wenfang Zhao, Yuan Mao, Yaguang Zhang

**Affiliations:** 1 Department of Biopharmaceutics, College of Food Science and Technology, Shanghai Ocean University, Shanghai, PR China; 2 Institut für Laboratoriumsmedizin, Charité-Universitätsmedizin Berlin, Campus Virchow Klinikum, Berlin, Germany; Consejo Superior de Investigaciones Cientificas, SPAIN

## Abstract

Lack of capacity to cross the nucleus membrane seems to be one of the main reasons for the lower transfection efficiency of gene vectors observed in vivo study than in vitro. To solve this problem, a new non-viral gene vector was designed. First, a degradable polyethylenimine (PEI) derivate was synthesized by crosslinking low-molecular-weight (LMW) PEI with N-octyl-N-quaternary chitosan (OTMCS), and then adopting a designed trifunctional peptide (RGDC-TAT-NLS) with good tumor targeting, cell uptake and nucleus transport capabilities to modify OTMCS-PEI. The new gene vector was termed as OTMCS-PEI-R18 and characterized in terms of its chemical structure and biophysical parameters. Gene transfection efficiency and nucleus transport mechanism of this vector were also evaluated. The polymer showed controlled degradation and remarkable buffer capabilities with the particle size around 100–300 nm and the zeta potential ranged from 5 mV to 40 mV. Agraose gel electrophoresis showed that OTMCS-PEI-R18 could effectively condensed plasmid DNA at a ratio of 1.0. Besides, the polymer was stable in the presence of sodium heparin and could resist digestion by DNase I at a concentration of 63U DNase I/DNA. OTMCS-PEI-R18 also showed much lower cytotoxicity and better transfection rates compared to polymers OTMCS-PEI-R13, OTMCS-PEI and PEI 25 KDa in vitro and in vivo. Furthermore, OTMCS-PEI-R18/DNA complexes could accumulate in the nucleus well soon and not rely on mitosis absolutely due to the newly incorporated ligand peptide NLS with the specific nuclear delivery pathway indicating that the gene delivery system OTMCS-PEI-R18 could reinforce gene transfection efficiency in vivo.

## Introduction

Cancer remains a global health problem and a major cause of death worldwide. Statistical analysis published by the International Agency for Research on Cancer from WHO reveals that if the estimated trends continue, the incidence of all cancer cases will raise from 12.7 million new cases in 2008 to 21.2 million by 2030 [1]. Facing such an alarming disease progression, varieties of therapies have been developed but expected efficiency has still not been reached. Cancer is a genetic disease induced by many factors [2]. The process of neoplastic cell apoptosis and its regulation and control factors are the hotspot of investigation in recent years. Gene therapy is tried to be realized through inducing cell apoptosis [3]. On one hand, gene therapy is considered to be a safe and effective treatment by using exogenous nucleic acids as therapeutic agents, which has the potential to depress oncogenes and restrain the proliferation of intractable tumors and has no effect on normal cells. However, the easy degradation in biological fluids and the low cellular uptake efficiency of nucleic acids cause gene therapy strongly subject to the development of gene vectors [4]. All in all, gene transfection is the key technique of gene therapy. At present, more and more attention is focused on non-viral vectors [5]. Polyethylenimine (PEI) is a kind of typical cationic polymer which has large amount of positive charge can form condensed complex with DNA spontaneously through electrostatic interactions. Due to its highly effective gene transfection, PEI has gained a remarkable interest in the field of non-viral gene delivery system [6–8]. However, testing of PEI vectors in preclinical studies identified several drawbacks: the contradiction between toxicity and transfection efficiency, lacking of specific tumor targeting ability, low transfection capacity in vivo.

Chitosan is a natural cationic non-toxic polysaccharide, which has been regarded as a fascinated candidate for transfection as it is biodegradable and biocompatible [9]. This attribute has been fully reflected in chitosan-mediated gene transfection systems. However, several problems need to be solved before practical application such as insolubility at physiological pH, and low transfection efficiency in particular [10].

It has been proved that PEI derivatives obtained by cross-linking low-molecular-weight (LMW) PEI with degradable materials display higher transfection efficiency and lower cytotoxicity [11,12]. To salve the conflict between the high cytotoxicity and low transfection efficiency of gene vector, a new polymer has been synthesized by covalent linkage between the activated chitosan OTMCS and non-toxic low molecularPEI (2 KDa) firstly, and was named OTMCS-PEI. As what we have reported in that paper, OTMCS-PEI revealed lower cytotoxicity for the property of degrading into small molecules after uptake into the cells. However, the transfection efficiency of the polymeric gene vector is not high enough and it was worth improving its tumor targeting [13].

The strategy for incorporating ligand peptide with favorite characteristics into target molecules has been the most considerable method in developing target specific non-viral gene delivery systems [14]. Integrin *αvβ3* receptor is highly expressed in tumor cells whereas rarely detectable on quiescent blood vessels. This character allows for its use as a suitable target for gene vector. Knowing that arginine-glycine-aspartate-cysteine (RGDC) peptide is a key binding moiety that has been shown to bind specifically to αvβ3 integrin receptors [15,16].

Cell membrane prevents xenobiotic from entering unless an active transport mechanism involved, which happens mainly with specific peptides. TAT peptide is one of the protein-derived peptides, which has been reported capable of facilitating the cellular uptake of macromolecular particles. The commonly used transduction domain of TAT extends from residues 47–57: Arg-Lys-Lys-Arg-Arg-Gln-Arg-Arg-Arg [17,18]. Therefore, we used RGDC peptide conjunction with TAT to yield a new chimeric peptide RGDC-NLS (named R13), and bifunctional peptide R11 to modify PEI derivates respectively, in order to improve cell selection, promote cargo transport and enhance transfection efficiency.

The polymer OTMCS-PEI-R13 has showed low cytotoxicity as OTMCS-PEI, and higher transfection in vitro. With the following in vivo studies, gene transfection efficiency was found to be much lower compared with in vitro. The main reason is that the transport manner of complexes across nucleus membrane in vivo is different from in vitro. DNA complexes get into nucleus via mitosis in vitro as tumor cells are in division phase, while most of the target cells in vivo are non-dividing, which makes it more difficult for gene transport. Lack of capacity to cross the nucleus membrane seems to be the principle cause of the low transfection efficiency observed in vivo study [19].

It is one of the major limitations for plasmid DNA to entry into the nucleus from the cytoplasm. Several groups have demonstrated that the attachment of a nuclear localization sequence (NLS) either directly to DNA or to polymers that form complexes with DNA lead to an increased nuclear import of DNA [20,21], especially in vivo tranfection with the non-dividing cells, resulting in enhanced protein expression. Talsma has declared that PEI conjugated to *σ1*-NLS performed substantially better as a transfection agent than PEI alone. With *σ1*-NLS, 69% of the internalized protein was recovered in the nuclear fraction after 6 h compared to just 10% when using unmodified *σ1*. At the same time, transfection of cells with PEI-*σ1*-NLS-DNA complexes resulted in an average 16-fold increase in luciferase activity compared to cells transfected with PEI-DNA. These results suggested that *σ1*-NLS is an efficient targeting ligand for non-viral gene delivery system based on PEI [22]. Macromolecule mediated by NLS is actively transported to nucleus via nuclear pore complex (NPC), and NLS of SV40 T-antigen with the major functional sequence Lys-Lys-Lys-Arg-Lys (KKKRK) was discovered firstly and has been the major research object. Therefore, we used the linear peptide R13 (RGDC-TAT), in conjunction with NLS to yield a novel peptide, RGDC-TAT (49–57)-NLS, named R18. The trifunctional R18 can adhere to tumor cells by targeting to *αvβ3* receptors, facilitate the cellular uptake and nuclear transport of polyplexes. Then we adopted R18 to modify OTMCS-PEI to prepare a new non-viral gene vector (OTMCS-PEI-R18). The new gene vector was characterized in terms of its chemical structure and biophysical parameters. Gene transfection efficiency of this vector was validated by in vitro and in vivo studies. Chemical inhibitors specific to various intracellular pathways and confocal laser scanning microscopy have been applied to investigate its nuclear transport. The purpose of the present study was to find a gene vector with low cytotoxicity and high transfection efficiency in vivo.

## Materials and Methods

### Materials

#### Chemicals

Branched PEI 2KDa, 3-(4, 5-dimethyl-2-thiazolyl)-2, 5-diphenyl tetrazolium bromide (MTT) and heparin were purchased from Sigma-Aldrich. Chitosan 2KDa (degree of deacetylation, 90%), dichloromethane, benzene, triethylamine, triphosgene, ethyl acetate, N-hydroxysuccinimide and absolute ethyl alcohol were purchased from Sinopharm Chemical Reagent Co, Ltd. DNase I was obtained from the Worthington Company. Dialysis bag (MWCO 7000) was purchased from Spectrum Chemical & Instruments. Paclitaxel (PTX), Hochest 33258 and fluorescein isothiocynate (FITC) were purchased from Sigma Aldrich. 4% paraformaldehyde was provided by Beijing Solarbio Science & Technology Co. Ltd. Colchicine (Col) and acrylamide were purchased from Beijing Hotaibio Science & Technology Co.

#### Plasmids

The plasmid encoding the enhanced green fluorescent protein (pEGFP-N2) was kindly supplied by Tongji University. The purity of the purified and concentrated DNA was determined by measuring its UV absorbance value at 260 nm and 280 nm respectively. Luciferase assay system for transfection and pGL3-Control vector modified with SC-40 promoter and enhancer encoding firefly luciferase were obtained from Promega Corp. The plasmids were amplified by Escherichia *coli* DH*5α* and prepared using the Tiangen End-free Plasmid Mega Kit according to the manufacturer’s specifications.

#### Peptide

The oligopeptide Arg-Gly-Asp-Cys-Arg-Lys-Lys-Arg-Arg-Gln-Arg-Arg-Arg-Lys-Lys-Lys-Arg-Lys (R18, MW 2439.99 Da) was synthesized by GL Biochem (Shanghai, China), and the amino acid sequence and purity were detected by mass spectroscopy and high performance liquid chromatography.

#### Cell culture

The Hela cell line was purchased from the Type Culture Collection of the Chinese Academy of Sciences, Shanghai, China. Culture medium (RPMI 1640) and fetal bovine serum (FBS) were purchased from GIBCO.

#### Animals

Athymic nude mice (males, 4–6 weeks old, 16–18 g) were purchased from the National Rodent Laboratory Animal Resources, Shanghai Branch (Shanghai, China). Animals were housed in a barrier facility on a high efficiency particulate arrestance (HEPA)-filtered rack under standard conditions of 12-hour light/dark cycles. The animals were fed an autoclaved laboratory rodent diet. All mouse surgical procedures were performed with the animals anesthetized by intramuscular injection of 50% ketamine, 38% xylazine, and 12% acepromazine maleate (0.02 ml). The maximum tumor size was less than 2 cm. The condition of the animals was monitored every day. Some animals did become severely ill and even died after being injected with Hela cells for establishing tumor model. These animals that became severely ill were treated with high concentration of carbon dioxide for euthanasia. The animals were all sacrificed 24 hours after giving doses. CO_2_ inhalation was used for euthanasia. To ensure death following CO_2_ asphyxiation, cervical dislocation was performed. All animal procedures were approved by the Committee for Animal Research of Shanghai Ocean University, China (SCXK (HU) 2007–0003) and carried out according to the Guide for the Care and Use of Laboratory Animals.

### Synthesis and characterization of OTMCS-PEI-R18

OTMCS and OTMCS-PEI were synthesized according to the method described in our previous work. Polymer OTMCS-PEI was conjugated with peptide using SMCC as crosslinker. Firstly, dimethyl sulfoxide (DMSO) was used as stock solution to prepare 3.33 mg/mL SMCC solution, and then the prepared solution was added dropwise into OTMCS-PEI solution (10 mg/mL in PBS) at 2:1, 10:1 mole ratios with constant stirring at room temperature for 30 min.

After complete reaction, the excessive non-conjugated crosslinker was removed by gel chromatography (Sephadex G-25, Pharmacia, Milton Keynes, UK) that had been equilibrated with PBS (0.1M, pH 7.4). Then, trifunctional peptide R18 solution (using 0.1 M PBS as solvent to10 mg/mL) was added dropwise into prepared OTMCS-PEI solution with gently stirring at 4°C overnight in the dark, R18 was reacted with the maleimide-actived OTMCS-PEI at a molar ratio of 2:1 and 10:1. The product was obtained by lyophilization after removing the excessive unreacted R18 by ultrafiltration with Amicon Ultra-4 centrifugal filter devices (Millipore, Billerica, MA). Finally, two types of cationic polymer were obtained and named OTMCS-PEI-R18-l and OTMCS-PEI-R18-h, respectively.

The structure of OTMCS-PEI-R18 was characterized by ^1^H nuclear magnetic resonance (^1^H NMR) spectra. 10 mg of OTMCS-PEI-R18 was dissolved in 0.6 mL of deuterium oxide (D_2_O) in an NMR tube, and the ^1^H NMR spectrum was recorded using a Varian 300 MHz spectrometer (Varian Medical Systems Inc, Palo Alto, CA) at room temperature.

### Degradation of OTMCS-PEI-R18

Degradation of OTMCS-PEI-R18 was estimated by the measurement of molecular weight at different time. 0.5 g of the polymers were dissolved in 10 mL of PBS (0.1M, pH 7.4) and incubated at 37°C with shaking at 100 rpm for different time, then solutions of the polymers were lyophilized and the molecular weights of lyophilized samples were measured by GPC-MALLS with 690 nm laser wavelength using TSK-GEL G5000PW_xL_ (temperature 30°C, pressure 1.58Mpa) operated at a flow rate of 0.5mL/min.

### Buffering capacity of the OTMCS-PEI-R18 polymer

The synthesized OTMCS-PEI-R18 polymer solution was prepared in a 50 mL flask (0.2 mg/mL, 30 mL), using pure water as control. After adjusting the initial pH to 10.0 with 0.1 M NaOH, 25μL increment of 0.1 M HCl was added, and the pH of the solution was measured with a pH meter after each addition [23].

### Particle size and Zeta potential measurements

Particle size and zeta potential of OTMCS-PEI-R18/DNA complexes were measured by an electrophoretic light-scattering spectrophotometer (Zetasizer Nano ZS90, MAN0317 Issue 5.0, Malvern Instruments Ltd. Malvern,UK), with a 90° scattering angle in PBS buffer at room temperature. The complexes were prepared at desired w/w ratios ranging from 5 to 40 with incubating for 30 min at room temperature, and then measured for size and zeta potential. All the experiments were performed in triplicate.

### Agarose gel retardation assay

The DNA complex ability of OTMCS-PEI-R18 was confirmed by agarose gel retardation experiment. Polymer/DNA complexes at different weight ratios were prepared with the plasmid DNA concentration of 25 ng/μL. Then applied mixed liquor of 5 μL of complexes solution and 1 μL of 5× loading buffer to 1% agarose gel. After electrophoresis was carried out in Tris-Acetate-EDTA (TAE) buffer at 160 V for 30 min, the gel was stained with 0.5 μg/mL ethidium bromide for about 10 min at room temperature and illuminated by an UV illuminator to show the location of the DNA.

### Resistance to DNase I digestion

Adding different amount of DNase I to 10 μL of polymer/DNA complexes solution (250 ng of plasmid DNA) in 0.5 mL Eppendorf tubes and incubated at 37°C for 30 min. Then adding 2 μL of EDTA (250 mM) and incubated at room temperature for 10 min to inactivate the enzyme. Finally, 10 μL of sodium heparin (2 mg/mL) was added and incubated at room temperature for 2 h to dissociate the complexes. After all of these steps, electrophoresis was conducted as the method above to evaluate the resistance to DNase I digestion.

### Resistance to sodium heparin

10 μL of polymer/DNA complexes solution (250 ng of plasmid DNA) was prepared in 0.5 mL eppendorf tubes, 2 μL of sodium heparin solution of different concentration was added to the tubes. After incubation at 37°C for 60 min (30 min for the latter), electrophoresis was conducted to evaluate the resistance of OTMCS-PEI-R18 to sodium heparin.

### Cell lines and culture conditions

Hela (cervical cancer line, adherent) were grown in RPMI 1640 medium (Gibco) supplemented with 10% (v/v) FBS. They were incubated at 37°C in a humidified atmosphere of 5% CO_2_.

### Cytotoxicity assay

The cell viability of Hela cells after treatment with OTMCS-PEI-R18 were determined by MTT assay [24]. The cells were seeded at a density of 5000 cells per well with 200 μL of growth medium in 96-well plates and incubated for 24 h to reach 80% confluence. Thereafter, they were treated with different concentrations of polymer (4 μg/mL, 8 μg/mL, 16 μg/mL, 24 μg/mL, 32 μg/mL) dissolved in fresh serum-free medium. 4 h later, the medium was replaced by 200 μL of fresh growth medium. After 72 h incubation, the medium was removed and 20 μL of MTT solution (5 mg/mL) and 180 μL of fresh growth medium were added to each well and incubated for 4 h at 37°C in a CO_2_-incubator. Then medium was removed and 150 μL of DMSO was added to dissolve any formazon crystals formed. Control groups were used in which cells without any treatment (culture medium + cells). To evaluate cell viability, the absorbance value at 490 nm and 570 nm was measured by an ELISA plate reader with background subtraction. The cell viability (%) was calculated according to the following equation:

Cell viability (%) = (*A*_test_/*A*_control_) × 100 (mean ± standard deviation, n = 6)

Where *A*_test_ is the absorbance of OTMCS-PEI-R18 treated cells and *A*_control_ is the absorbance of the untreated cells.

### In vitro gene transfection

The plasmid pEGFP-N2 and pGL3-Control were used to measure the gene transfection efficiency of OTMCS-PEI-R18. Hela cells were cultured in 24 well plates with medium containing 10% FBS. Cells were grown to 80% confluence before transfection. For transfection, cells were washed twice with PBS and exposed to polymer/DNA condensates suspended in medium without FBS, the weight ratio of polymer and DNA was set as 5, 10, 20, 30 (2.5 μg of plasmid DNA per well). Accordingly, the concentrations of polymers were 12.5 μg/mL, 25 μg/mL, 50 μg/mL, 75 μg/mL respectively. The plates were incubated at 37°C for 4 h. Then the wells of plates were rinsed thoroughly with PBS and filled with 1 mL of culture medium with 10% FBS and incubated at 37°C for 48 h. After transfection for 48 h, the pEGFP-N2 expression was observed with an inverted fluorescent microscope (AE-31, Motic Corporation, Wetzlar, Germany). Luciferase gene expression was measured by a luminescence assay. After transfection for 48 h, the culture medium was discarded and cell lysate was harvested after gentle shaken of cells in 200 μL of cell culture lysis reagent (CCLR) for 15 min at room temperature. The lysate was centrifuged for 5 min at 14,000 rpm at 4°C. 20 μL of supernatant was diluted into 100 μL of luciferase reaction buffer (Promega) and luciferase activity was measured with a luminometer (Turner Designs Luminometer Model TD-20/20, Promega). The relative light units (RLUs) were normalized against protein concentration in the cell extracts, which was measured using a BCA protein assay kit (Pierce, Rockford, IL) [25].

### Determination of confocal laser scanning microscopy

The in vitro cellular uptake was determined by confocal laser scanning microscopy. OTMCS-PEI-R18 was labeled with FITC firstly. Hela cells were seeded into six-well plates within cell coverslip pre-paved at a density of 5×10^5^ cells per well and incubated for 12 h to obtain approximately 60% cell confluence. Then the cells were incubated with 1640 medium containing FITC-labeled OTMCS-PEI-R18 /DNA complexes for 0.5 h, 1 h and 2 h respectively. After that, the medium was removed and cells were fixed with 4% paraformaldehyde for 30 min and stained with 10 μg/mL Hochest 33528 for 15 min. Cells were rinsed 3 times with PBS after every step to remove the surface-associated complex and free reagent. Subsequently cell coverslip was placed on microscope slide with a drop of Anti fluorescent quenching agent, and the intracellular distribution of complexes was analyzed by confocal laser scanning microscope (CLSM, Olympus, FV1000-IX81,Japan) [26–28].

### Nuclear transport capability

Chemical inhibitors have an enormous impact on the intracellular components and structure, and then affect the transfection processing of complexes. PTX is initially characterized as a mitotic inhibitor, and its anti-neoplastic effect is derived from binding to tubulin and excessive microtubule stabilization. PTX is immunostimulatory against tumors and also regulates lymphocyte activation suggesting that apart from promoting inhibition in cell division [29]. Colchicine principally is a microtubule inhibitor, thus prevents cell migration, division, and polarization [30]. Acrylamide is a known disrupter of intermediate filaments [31].

In this study, Hela cells were treated with microtubule inhibitors Colchicine (Col), Paclitaxel (PTX) and intermediate filament inhibitor Acrylamide to inhibit cell division, gene transfection efficiency decreased as the DNA complexes couldn’t get into nucleus via cell division. The impacts of these inhibitors on transfection efficiency of OTMCS-PEI/DNA, OTMCS-PEI-R13/DNA and OTMCS-PEI-R18/DNA were different as the distinguishing features of the ligand being modified, which reflected the nucleus transport capability of the ligand. The effects of these inhibitors were investigated using the pGL3-Control reporter gene. All of the experiment methods refer to 2.10, except for the difference that before transfection, the culture medium was replaced with 400 μL of serum-free RPMI 1640 medium containing different concentrations of inhibitors and incubated for 30 min.

### In vivo gene transfection

Athymic nude mice (males, 4–6 weeks old, 16–18 g) were used for detecting gene transfection efficiency of OTMCS-PEI-R18, OTMCS-PEI-R13, OTMCS-PEI in vivo. These mice were divided into five groups (5 mice per group). 2 × 10^6^ Hela cells suspended in 0.2 mL of physiological saline were injected subcutaneously into each mouse to develop tumor models [32,33]. Two weeks later, when the mean longitudinal diameters of the subcutaneously transplanted tumor reached 10 mm, 250 μL of sterile polymer/DNA complexes solution containing 30 μg of the pGL3-Control reporter gene was injected into the tail vein of each mouse. Detailed operation are as followed: Group 1, OTMCS-PEI-R18-l/DNA complexes, w/w 30. Group 2, OTMCS-PEI-R18-h/DNA complexes, w/w 30. Group 3, OTMCS-PEI-R13/DNA complexes, w/w 10. Group 4, OTMCS-PEI/DNA complexes, w/w 30. Group 5, PEI 25 KDa/DNA complexes, w/w 30.

24 hours later, 5 mice of each group were sacrificed by euthanasia, the major tissues (heart, liver, spleen, lung, kidney) and the subcutaneously transplanted tumors were removed. These tissues and tumors were collected, ground and homogenized in cell culture lysis reagent (CCLR). The cell lysate was centrifuged for 10 min at 9,800 rpm. Luciferase activity was measured with a luciferase assay kit in a signal-well luminometer. The RLUs were detected against protein concentration in the cell tissue extracts, which was measured with the a BCA protein assay kit (Pierce, Rockford, IL).

## Results and Discussion

### Synthesis and characterization of OTMCS-PEI-R18

**Fig 1:**
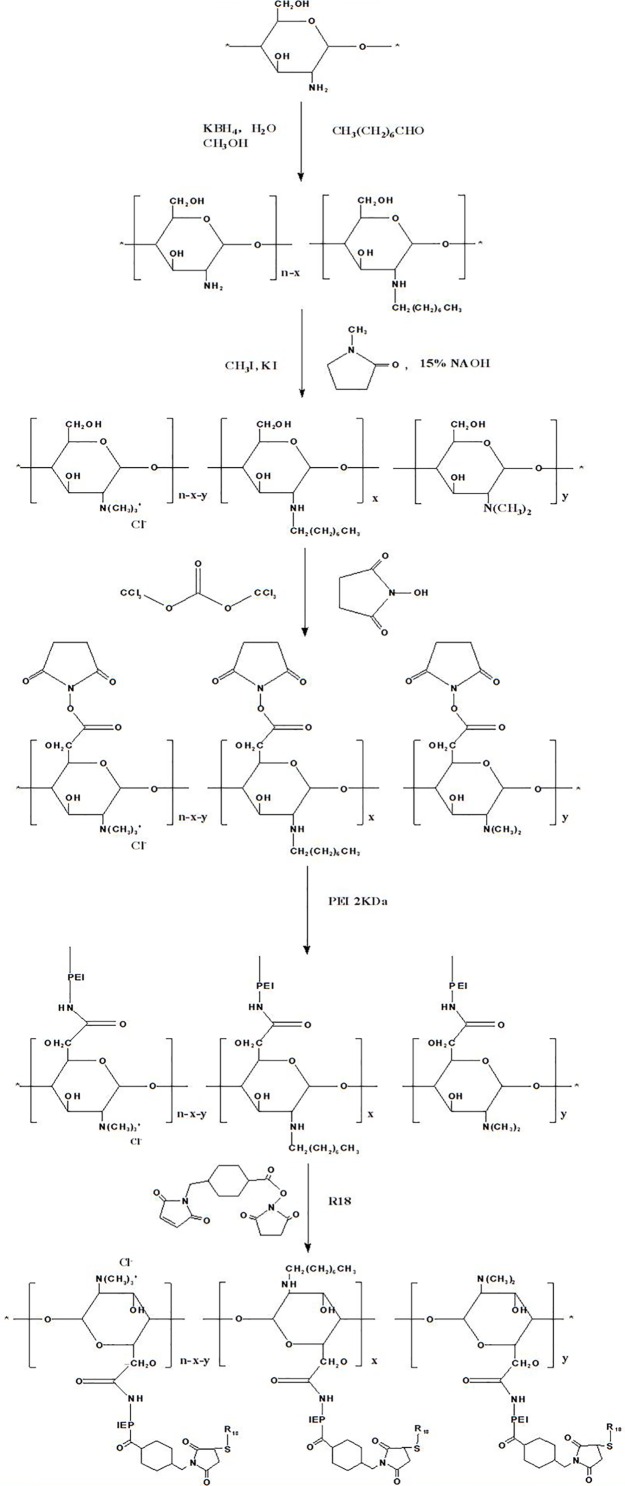
Synthesis and putative structure of OTMCS-PEI-R18.

The degradable PEI derivates of OTMCS-PEI were synthesized by linking PEI 2 kDa with OTMCS and then conjugated with a trifunctional peptide R18 to prepare a new nonviral gene delivery vector OTMCS-PEI-R18 (Fig 1). As for OTMCS-PEI, the free hydroxyl groups of OTMCS were activated by succinimidyl carbonate in advance and then linked to the amino groups of PEI. SMCC was used as a crosslinker to conjugate OTMCS-PEI with R18.

**Fig 2:**
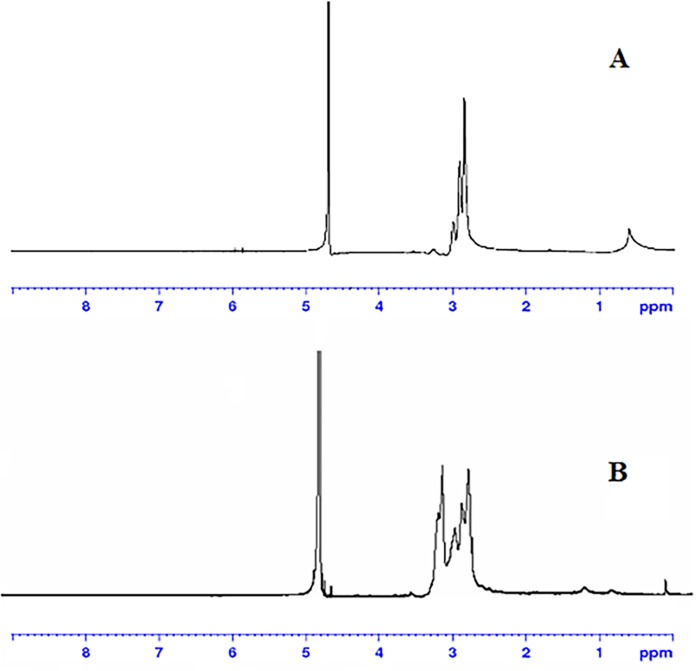
^1^H NMR spectra of (A) OTMCS-PEI and OTMCS-PEI-R18.

Fig 2A exhibited the ^1^H NMR spectra of OTMCS-PEI in D_2_O, where the glucosamine proton peaks appear at 3.136 ppm. Fig 2B exhibited the ^1^H NMR spectra of OTMCS-PEI-R18 in D_2_O. Comparing OTMCS-PEI with OTMCS-PEI-R18, the proton peaks of OTMCS-PEI-R18 moved to the lower magnet field due to the production of the groups with electronic screening effect from R18. The performances of characteristic peaks have changed, the proton peaks at 2.719–2.824 ppm depressed and the peak value at 1.422 ppm decreased indicating that OTMCS-PEI-R18 was successfully prepared.

**Fig 3:**
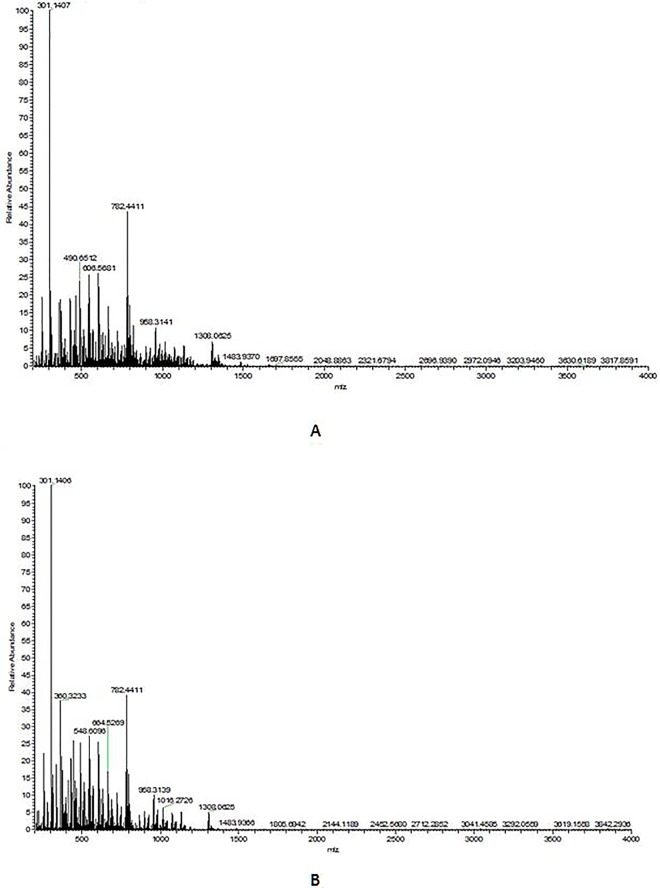
MALDI-TOF mass spectra of (A) OTMCS-PEI and (B) OTMCS-PEI-R18.

OTMCS-PEI and OTMCS-PEI-R18 were further characterized using MALDI-TOF (Fig 3A and 3B). Due to the high polydispersity of our polymers and the resulting mass discrimination against high-mass oligomers in the MALDI-TOF spectra [34], the molecular weight distribution of OTMCS-PEI and OTMCS-PEI-R18 can hardly be accurately determined using the results. However, compared with the position of the peaks in the spectrum of OTMCS-PEI, peaks in the spectrum of OTMCS-PEI-R18 have been shifted towards the higher mass rang. Such a shift indicated that the molecular mass of OTMCS-PEI-R18 is higher than OTMCS-PEI, suggesting successful cross-linking of OTMCS-PEI with R18 by SMCC.

### Buffering capacity of the OTMCS-PEI-R18 polymer

**Fig 4:**
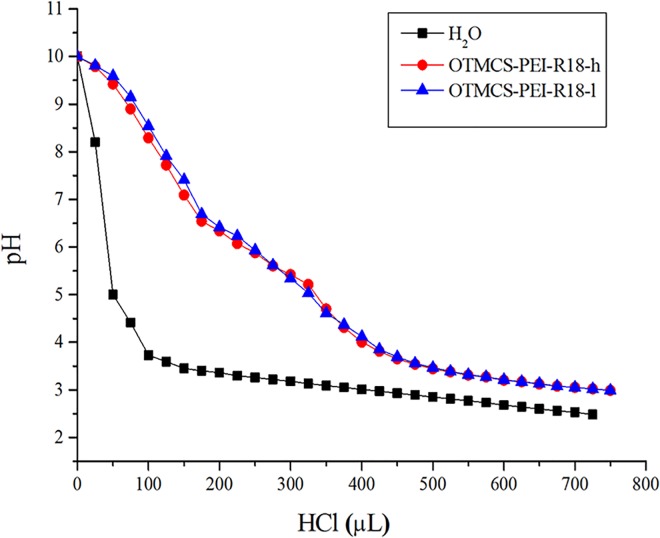
Determination of buffering capacity of the OTMCS-PEI-R18 polymer by acid-base titration. Solution containing the polymer (0.2 mg/mL) was adjusted to pH 10.0, and then titrated with HCl from 10.0 to 3.0.

The successful escape of plasmid DNA from the endosome of cell is the premise of transfection, in consequence, the buffering capacity of the gene vector related to the escape process is of great concern. Fig 4 showed that compared with pure water the polymer OTMCS-PEI-R18 had relatively high buffer capability especially in the pH range of 3–7. The proton sponge of the polymers ensures buffering inside the endosome, resulting in the degradation of lysosome so that the DNA can be released in time. OTMCS-PEI-R18 is sufficiently suitable for gene transfection.

### Particle size and Zeta potential measurements

**Fig 5:**
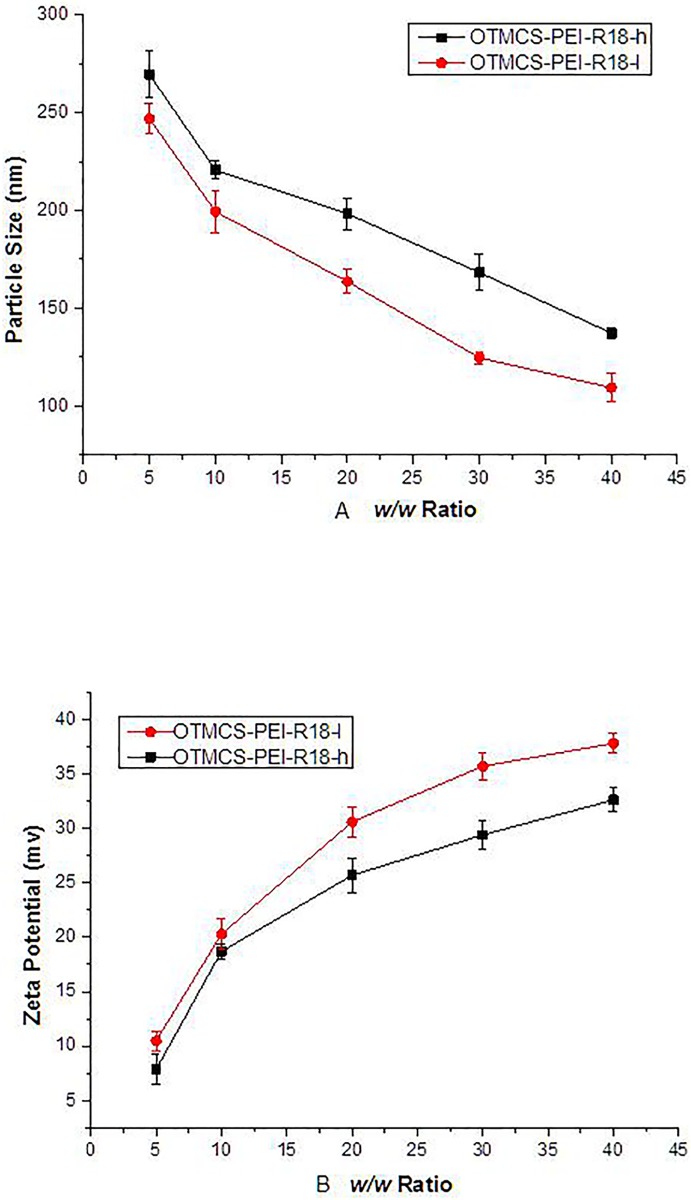
Particle size and Zeta potential of OTMCS-PEI-R18/DNA at various w/w ratios. The average diameter gradually decreased (A) and the Zeta potential gradually increased (B) as the ratio of conjugated DNA increased.

A successful gene delivery system requires that DNA must be condensed by polycation into nanoparticles small enough to facilitate cellular uptake [32]. The particle size and zeta potential of OTMCS-PEI-R18/DNA were measured at different w/w ratios. As shown in Fig 5A, the particle size of complexes OTMCS-PEI-R18/DNA decreased as the increasing of w/w ratio. Both polyplexes could condense DNA with a mean size of 100–300nm, which is suitable for efficient gene delivery.

The surface charges on the polymer/DNA nanoparticles were represented by the zeta potential, and a suitable cationic charge on the nanoparticles can electrostatically intect with anion charged cellular membranes to promote complexes enter cells. As Fig 5B showed, the zeta potential of OTMCS-PEI-R18/DNA complexes increased from 5 to 40 mV with an increasing of w/w ratio. In addition, OTMCS-PEI-R18-l had a higher zeta potential as compared with the other polymer at any designed ratio, probably because of the linking of less peptides.

### Condensation status of plasmid DNA by OTMCS-PEI-R18

**Fig 6:**
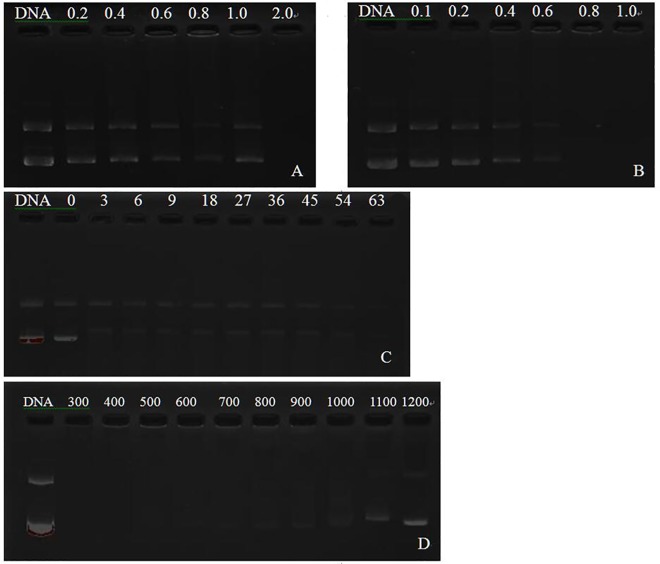
Agarose gel electrophoresis of plasmid DNA and OTMCS-PEI-R18 /DNA complexes. Agarose gel electrophoresis of OTMCS-PEI-R18-h/DNA(**A**) and OTMCS-PEI-R18-l/DNA(**B**) complexes at various w/w ratios, respectively. (**C**) Protection of plasmid DNA from degradation by DNase I at different concentrations of 0, 3, 6, 9, 18, 27, 36, 45, 54 and 63 U DNase I/μg DNA. (**D**) Protection of plasmid DNA from dissociation by sodium heparin at varying concentrations of 300, 400, 500, 600, 700, 800, 900, 1000, 1100, 1200 μg/mL.

Agarose gel retardation experiments was adopted to determine DNA condensation capacity of OTMCS-PEI-R18. DNA condensates were prepared at a DNA concentration of 25 ng/μL with polymers at various weight ratios. Complexes solutions were remained at 4°C for 30 min before electrophoresis. As it shown in Fig 6A and 6B, the movement of the plasmid DNA in the gel was blurry and retarded as proportion of the polymers increased due to the polymers bind to DNA and neutralize its charge. When DNA was coated by cationic polymer completely, the complexes would be electroneutral or even electropositive and stop shifting to the anode. In this experiment, the polymer of OTMCS-PEI-R18-l was able to condense DNA at a w/w ratio of 0.8 effectively while the other polymer of OTMCS-PEI-R18-h was at a w/w ratio of 2.0.

### Protection of OTMCS-PEI-R18 on plasmid DNA

Considering the complex environment in vivo, protection of loaded DNA against degradation is the premise of efficient transfection. So the stability of OTMCS-PEI-R18/DNA (w/w ratio of 20) condensates was determined by incubating them in DNase I and sodium heparin with increasing concentrations in vitro, respectively. After exposed to DNase I, the DNA protected by polymer was released by sodium heparin. As the Fig 6C showed, polymer could protect OTMCS-PEI-R18/DNA condensates from enzymatic DNase I digestion well, even when the concentration up to 63 U DNase I/μg DNA, partial DNA was still protected. Under the same experimental conditions, naked DNA was digested by DNase I at the concentration of 0.08 U DNase I/μg DNA [35].

Sodium heparin was used to simulate molecules with negative charges in vivo, which dissociate cationic polymer with its negative charges. As Fig 6D showed, plasma DNA from polymer/DNA condensates remained undetectable by agarose gel electrophoresis when incubated with sodium heparin at a concentration lower than 500 mg/mL. OTMCS-PEI-R18 /DNA were not completely dissociated until the concentration exceeded 1200 mg/mL.

These results demonstrated that OTMCS-PEI-R18 protected DNA from dissociation by DNase I and negative charged molecular.

### Degradation and Cytotoxicity assay

**Fig 7:**
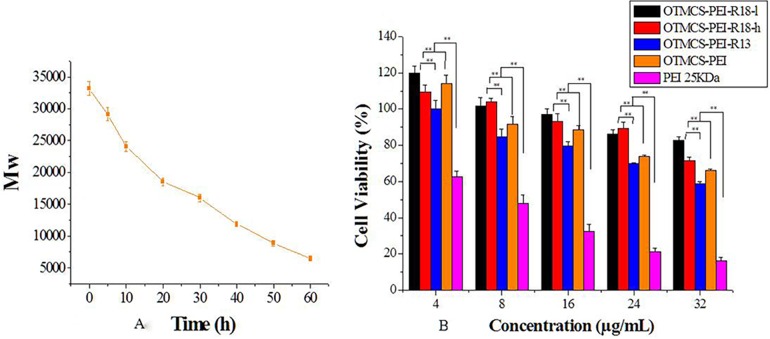
The cytotoxicity of OTMCS-PEI-R18. **(A)** Degradation of OTMCS-PEI-R18. **(B)** Cytotoxicity of OTMCS-PEI-R18 and other PEI derivates at various concentrations in Hela cell line using the MTT assay. OTMCS-PEI-R13, OTMCS-PEI and PEI 25 KDa were used as controls.

The cytotoxicity was found to be affected by molecular weight of polymers, resulting that the HMW PEI was limited in transfection due to its high cytotoxicity. That is good degradation of gene delivery polymers in vivo is very important for safe gene transfection. The degradation ability of OTMCS-PEI-R18 was shown in Fig 7A. OTMCS-PEI-R18 was degraded slowly and the degradation was nearly completed after about 60 h.

The goal of our study was to proceed a gene delivery system with efficient transfection and low cytotoxicity. In this paper, cytotoxicity of OTMCS-PEI-R18 was analyzed in Hela cells by MTT assay, and OTMCS-PEI, OTMCS-PEI-R13, PEI 25 KDa were performed as controls. Fig 7B showed the cell viability after incubation with polymer for 72 h as the concentration increasing from 4 μg/mL to 32 μg/mL. Cell viability of OTMCS-PEI-R18 was much higher than PEI 25 KDa, and similar to OTMCS-PEI, OTMCS-PEI-R13 at any concentration (*P<*0.01). In addition, OTMCS-PEI-R18 showed litter cytotoxicity (cell viability remained > 60%) when at a high concentration as 32 μg/mL. At the same time, cell viability decreased as the concentration increase, demonstrating it’s a typically dose-dependent process, so the application of the polymer had better be controlled in a range of proper concentration. The low cytotoxicity of these three polymers may be attributed to the degradation of polymers under physiological conditions.

### In vitro transfection efficiency

**Fig 8:**
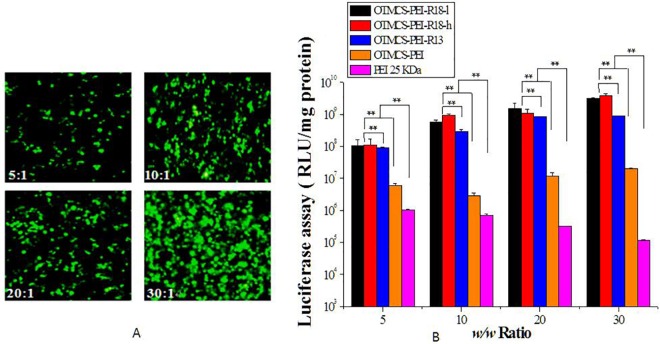
Gene transfection efficiency of OTMCS-PEI-R18/DNA polyplexes. **(A)** Representative fluorescence images for transfection in Hela cells using OTMCS-PEI-R18 at w/w ratios of 5, 10, 20, and 30. **(B)** Transfection efficiency of OTMCS-PEI-R18/DNA complexes in Hela cell line at w/w ratios of 5, 10, 20, and 30.

pEGFP-N2 and pGL3-Control were used to evaluate gene transfection of the newly synthesized OTMCS-PEI-R18 polymer. Fig 8A displayed representative fluorescence images of Hela cells transfected by OTMCS-PEI-R18/pEGFP-N2 at weight ratio range from 5 to 30. According to these images, the pEGFP-N2 reporter gene was effectively transfected into Hela cells. The percentage of green fluorescence positive cells was proportional to the w/w ratio of OTMCS-PEI-R18/DNA and the highest proportion of green fluorescence positive cells was observed at the weight ratio of 30.

Fig 8B showed the gene transfection efficiency of OTMCS-PEI-R18/pGL3-Control complexes in Hela cells in comparison with other polymers. OTMCS-PEI-R18/DNA complexes displayed much higher transfection level than other complexes at any designed weight ratio. The highest luciferase expression delivered by OTMCS-PEI-R18 was almost 5 times as OTMCS-PEI-R13, 10^2^ times as OTMCS-PEI and even 10^4^ times as PEI 25 KDa. OTMCS-PEI-R18-l and OTMCS-PEI-R18-h both revealed that maximal transfection efficiency at high weight ratio (almost at the concentration of 75μg/mL). Moreover, significant enhancement of luciferase expression was observed as the raw ratio of R18 increased, which demonstrated that the addition of R18 considerably enhanced the uptake of the polymer-delivered DNA.

### Nuclear transport evaluation

**Fig 9:**
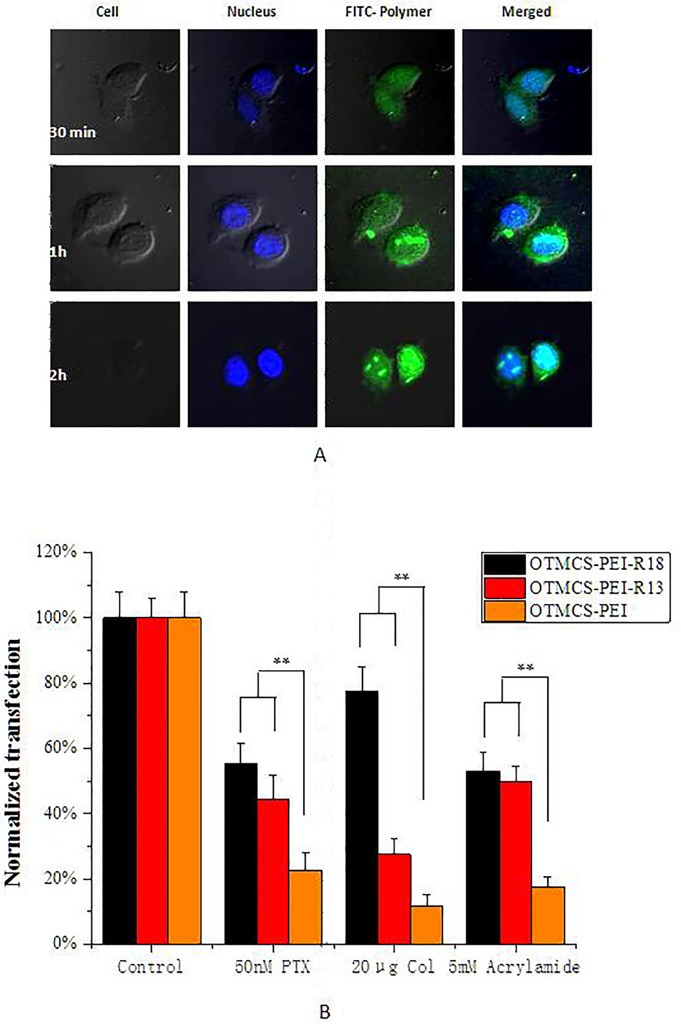
The intracellular distribution of complexes OTMCS-PEI-R18/DNA. **(A)** Fluorescent confocal microscopic images of Hela cells treated with FITC-labeled OTMCS-PEI-R18 polyplexes for 0.5 h, 1 h and 2 h, where the FITC-labeled OTMCS-PEI-R18 was shown in green, and the HE stained nuclear was shown in blue. **(B)** Inhibitory effect of different concentrations of Paclitaxel (PTX), Colchicine (Col) and Acrylamide on transfection efficiency of OTMCS-PEI-R18/DNA complex in Hela cells at w/w ratio of 30. The RLU/mg protein in control cells was set to 100%.

To visually reveal intracellular fate of OTMCS-PEI-R18 at different time points, Hela cells were treated with the FITC-labeled OTMCS-PEI-R18/DNA complexes. Here, DNA was non-functional plasmid used to help polymer to form the condensed nanoparticles. The results were shown in Fig 9A, FITC-labeled OTMCS-PEI-R18 was shown in green, the nuclear stained with HE was blue. After incubation for 30 min, mainly FITC-labeled OTMCS-PEI-R18/DNA complexes distributed in the cytoplasm and a few had entered into nucleus. At next time point for 1 h, large amount of green accumulated in the nucleus and it is obviously that, more green accumulation was observed in the nucleus treated for 2 h in comparison with treated for 1 h. Nuclear membrane is one of the barriers at gene delivery system which was needed to overcome for efficient transfection. It is proved that OTMCS-PEI-R18 could deliver plasmid DNA into nucleus well soon, which is prerequisite for gene therapy, and chemical inhibitors specific to microtubules and intermediate filaments were used to investigate whether it is due to the conjunction of NLS.

Microtubules and intermediate filaments are basic cytoskeletons of the cell. Microtubules play important roles in mitosis, and intermediate filaments are closely related to microtubules, might contribute to the stability and assembly of microtubules. Colchicine (Col) and paclitaxel (PTX) were used as inhibitors of microtubules. Col caused depolymerization of microtubules, but PTX was shown to bind to tubulin and stimulate excessive microtubules stabilization. Acrylamide was used to study intermediate filaments. All of them would disrupt the mitosis in cell division phase. OTMCS-PEI-R13, OTMCS-PEI were performed as controls. In Fig 9B, the effect of inhibition was more obvious for OTMCS-PEI and OTMCS-PEI-R13. Compared with the blank control groups, gene transfection efficiency of OTMCS-PEI without ligand modification decreased nearly 80%, and even up to 90% when treated with 20 μg Col. The inhibitory effect on transfection of OTMCS-PEI-R13 was weakened in the presence of TAT peptide, but still be suppressed by almost 50%. By contrast, the weakest inhibition was showed for OTMCS-PEI-R18 and the inhibitory rate was just about 20% when treated with 20 μg Col. It proved that the nuclear transport of OTMCS-PEI-R18/DNA may not rely on cell division completely and the introduction of NLS facilitated the transport process. DNA nanoparticles always get into the nucleus during mitosis of the cell in vitro, the introduction density of OTMCS-PEI-R13/DNA and OTMCS-PEI/DNA without modification of NLS sharply decreased after the inhibition of cell division, while OTMCS-PEI-R18/DNA complexes could transport into nucleus under the NLS mediated through NPC (nuclear pore complex), which is advantageous for gene transfection in vivo.

### In vivo transfection efficiency

**Fig 10:**
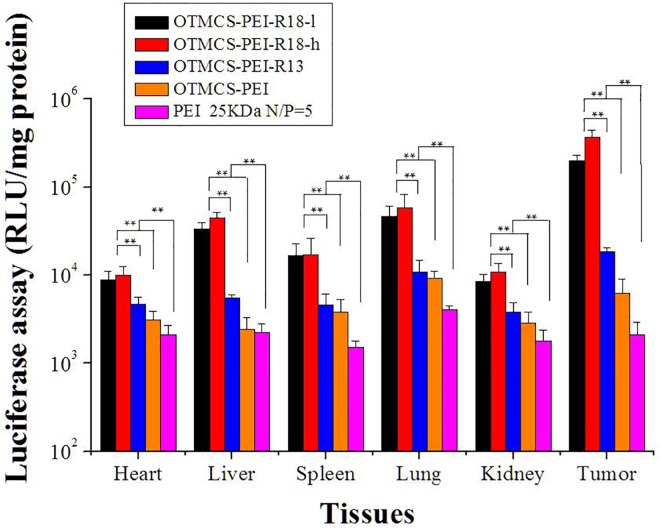
Transfection efficiency of pGL3-Control as a reporter gene in mice.

Efficient delivery system for therapeutic genes with least side effects would have great potential in clinical treatment. In order to research the delivery capacity of OTMCS-PEI-R18 in vivo, complexes (polymer/pGL3-Control) containing 30 mg of plasmid DNA prepared at w/w ratio of 30 were tail intravenously injected into Hela tumor-bearing nude mice, and OTMCS-PEI-R13, OTMCS-PEI, PEI 25 KDa were used as controls. As Fig 10, complex OTMCS-PEI-R18-marker in vivo gene transfection exhibited higher luciferase expression level in any tissues compared with OTMCS-PEI-R13, OTMCS-PEI and PEI 25 KDa. For PEI 25 KDa and OTMCS-PEI, expression level in tumor was lower than that in lung due to nonspecific adsorption, but with the modification of peptide R13, tumor expression of OTMCS-PEI-R13 enhanced and basically equal to lung, proving tumor targeting capabilities of pepetide R13. Further, significantly higher expression in tumor was observed after the introduction of NLS peptide, almost 12-fold increase in luciferase activity compared with OTMCS-PEI-R13. Gene expression of both OTMCS-PEI-R18-l/DNA and OTMCS-PEI-R18-h/DNA complexes was highest in the tumor, and OTMCS-PEI-R18-h showed higher luciferase expression. This phenomenon is absolutely consistent with the previous study, which further proved the ability of facilitating nucleus import of the new attached NLS peptide.

## Conclusion

We developed a new non-viral gene vector OTMCS-PEI-R18 by cross-linking LMW PEI with OTMCS and further coupled with a trifunctional peptide R18 for specific tumor targeting, cellular and nucleus transport. Through characterization of its chemical structure and biophysical parameters, the polymer OTMCS-PEI-R18-l could condense plasmid DNA to nanoparticle at the weight ratio about 0.8. In addition, the new polyplexes could efficiently DNA into stable nanoparticle with proper size and zeta potential. The nanoparticle could resist dissociation by DNase I at a concentration of 63U DNase I/DNA and dissociation induced by 1200 μg/mL sodium heparin, it also showed controlled degradation, high buffer capabilities and low cytotoxicity. These means the polymer absolutely proper as a gene vector for in vivo application. The gene vector system OTMCS-PEI-R18 displayed better transfection rates as compared to polymers OTMCS-PEI-R13, OTMCS-PEI and PEI 25 KDa in vitro and in vivo, due to the newly incorporated ligand peptide NLS with the properties of promoting nuclear delivery. Images got by a confocal fluorescence microscope proved that after taking up by the cells, the complexes accumulated in the nucleus eventually. In addition, the study on nuclear transport also proved that introduction of NLS facilitated the nuclear transport of OTMCS-PEI-R18/DNA. In conclusion, this new polymer OTMCS-PEI-R18 may act as promising candidates for non-viral gene delivery in future in vivo application with low cytotoxicity and high transfection efficiency. We are recently carrying out more comprehensive studies with the aim of exploring the nuclear transport pathway of OTMCS-PEI-R18.
